# A cross-sectional study of explainable machine learning in Alzheimer’s disease: diagnostic classification using MR radiomic features

**DOI:** 10.3389/fnagi.2023.1149871

**Published:** 2023-06-07

**Authors:** Stephanos Leandrou, Demetris Lamnisos, Haralabos Bougias, Nikolaos Stogiannos, Eleni Georgiadou, K. G. Achilleos, Constantinos S. Pattichis, Alzheimer’s Disease Neuroimaging Initiative

**Affiliations:** ^1^School of Sciences, European University Cyprus, Nicosia, Cyprus; ^2^University Hospital of Ioannina, Ioannina, Greece; ^3^Discipline of Medical Imaging and Radiation Therapy, University College Cork, Cork, Ireland; ^4^Division of Midwifery and Radiography, City, University of London, London, United Kingdom; ^5^Medical Imaging Department, Corfu General Hospital, Corfu, Greece; ^6^Metaxa Anticancer Hospital, Athens, Greece; ^7^Department of Computer Science and Biomedical Engineering Research Centre, University of Cyprus, Nicosia, Cyprus; ^8^CYENS Centre of Excellence, Nicosia, Cyprus

**Keywords:** Alzheimer’s disease, MRI, machine learning (ML), radiomic, explainability and interpretability

## Abstract

**Introduction:**

Alzheimer’s disease (AD) even nowadays remains a complex neurodegenerative disease and its diagnosis relies mainly on cognitive tests which have many limitations. On the other hand, qualitative imaging will not provide an early diagnosis because the radiologist will perceive brain atrophy on a late disease stage. Therefore, the main objective of this study is to investigate the necessity of quantitative imaging in the assessment of AD by using machine learning (ML) methods. Nowadays, ML methods are used to address high dimensional data, integrate data from different sources, model the etiological and clinical heterogeneity, and discover new biomarkers in the assessment of AD.

**Methods:**

In this study radiomic features from both entorhinal cortex and hippocampus were extracted from 194 normal controls (NC), 284 mild cognitive impairment (MCI) and 130 AD subjects. Texture analysis evaluates statistical properties of the image intensities which might represent changes in MRI image pixel intensity due to the pathophysiology of a disease. Therefore, this quantitative method could detect smaller-scale changes of neurodegeneration. Then the radiomics signatures extracted by texture analysis and baseline neuropsychological scales, were used to build an XGBoost integrated model which has been trained and integrated.

**Results:**

The model was explained by using the Shapley values produced by the SHAP (SHapley Additive exPlanations) method. XGBoost produced a f1-score of 0.949, 0.818, and 0.810 between NC vs. AD, MC vs. MCI, and MCI vs. AD, respectively.

**Discussion:**

These directions have the potential to help to the earlier diagnosis and to a better manage of the disease progression and therefore, develop novel treatment strategies. This study clearly showed the importance of explainable ML approach in the assessment of AD.

## 1. Introduction

According to World Health Organization (WHO), Alzheimer’s disease (AD) is in the top 10 diseases leading cause of death in the United States (US) and it cannot be prevented or cured (Vaz and Silvestre, 2020). It is the most common form of dementia and clinically the disease manifests as memory loss disorientation, confusion and behavior changes, whereas, in advance subjects there is difficulty in speaking, walking even swallowing, therefore, these individuals require 24/7 care. According WHO,^1^ there are 47 million patients worldwide and by 2030 this number is projected to increase to 78 million. Although, therapeutic guidelines of the disease are beyond the scope of this manuscript, interesting information regarding new therapeutic guidelines and potential benefits of electromagnetic fields (EMF) as an innovative approach for the treatment of AD have been reported to many studies (Ahmad et al., 2020; Fakhoury et al., 2021).

The diagnosis of the disease still remains probable and relies on clinical and neuropsychological tests (Folstein et al., 1975; Morris, 1993) which evaluate memory and language abilities. Therefore, a subject is categorized as a patient with “probable” AD and only post-mortem material will confirm the disease through the detection of deposits of amyloid-β (Aβ) plaque deposition and tau protein (NFTs) in the brain tissue (Braak and Braak, 1997). However, decades before the first clinical symptoms become apparent, there is an inevitable progression of atrophy, which initially affects the Medial Temporal Lobe (MTL) (Scahill et al., 2002; Petrella et al., 2003; Jack et al., 2004). Most importantly, mild cognitive impairment (MCI) which is the pre-dementia stage cannot be identified easily by cognitive tests, as these subjects do not have major memory problems which will affect their daily routine, therefore they cannot be detected. Thus, a large effort has been made to develop techniques that will allow the early identification of AD, and in particular in quantitative imaging.

In diagnostic imaging interpretation, radiologists describe qualitative characteristics of a region of interest (ROI) such as its size, shape, speculation, cavitation or contrast enhancement. The necessity of quantitative imaging in AD assessment derives from the fact that the human eye cannot perceive anatomical changes through qualitative imaging in the early stages of the disease, whereas, through radiomic unique information which may contain neurodegenerative changes can be extracted at the microscopic level and before atrophy of the brain occurs. Through quantitative imaging high-dimensional minable data (radiomics) are extracted, such as histogram, texture features, wavelets, Laplacian transforms, minkowski functionals or fractal dimensions. In medical imaging, radiomics refers to the extract of a large number of quantitative features to be used in the improvement of diagnosis, prognostication and decision support. Through radiomics valuable features (patterns) that are imperceptible to the human eye are extracted, providing the clinician with valuable information (Vial et al., 2018). The term radiomics, is motivated by the idea that biomedical images contain hidden information that reflects the underlying pathophysiology and that these relationships can be revealed through quantitative image analysis (Gillies et al., 2016). Features are specific image characteristic (patterns) that may not be visible to a human but are recognized by a computer algorithm. In combination with clinical data these models could provide better classification accuracy.

Radiomics were initially used to identify imaging biomarkers related to cancer (Mayerhoefer et al., 2020). However, nowadays are being used for the assessment of other diseases as well, such as AD (Chincarini et al., 2011; Feng et al., 2018; Leandrou et al., 2020). After the acquisition of high-quality images, the identification and segmentation of the ROIs is performed. Then, from these ROIs quantitative features are extracted to develop diagnostic or predictive models (Gillies et al., 2016). Brain magnetic resonance imaging (MRI) studies require preprocessing steps such as spatial registration and normalization, as mentioned in the section “2. Materials and methods.” Although volumetry represents the most used method to date, there is lack of research in the assessment of AD using texture analysis. The study of Sørensen et al. (2015), found that hippocampal texture was superior to volume reduction for the disease prediction. For a comprehensive read in the assessment of AD using quantitative methods, including texture, the reader is refer to Leandrou et al. (2018).

With the rapid development of the acquisition imaging techniques there is high dimensional and multimodal neuroimaging data available which is difficult to analyze with contemporary methods. As a result, the high demand of computational analysis, has evolved the use of computational machine learning (ML) methods for the integrative analysis of those data. ML can be used to determine which features alone or in combination are strongly correlated with outcomes for a disease. More importantly, ML techniques such as deep learning and other neural networks allow for the discovery of relationships that have not been considered within the radiomic feature set extracted (Vial et al., 2018), therefore, lead to new knowledge discovery of a complex disease.

Due to the plethora of information provided by radiomics, genetics and cognitive tests, AD research through ML methods is very popular. Table 1 tabulates studies that have used ML techniques and radiomics for the assessment of AD, proposing that these methods are suitable for the AD diagnosis. From the results high accuracy metrics are reported, however, in the literature there are many studies that used a very small sample, or do not refer to the preprocessing methods used or the split of train or testing set, showing that their methodology might not be appropriate.

**TABLE 1:** Selected quantitative MRI studies where machine learning (ML) techniques and radiomics were used in the assessment of AD.

References	Subjects	Description	Split	Methodology	Results
Bogdanovic et al., 2022	Total 9,592 subjects (NC, EMCI, LMCI, SMC. AD)	Structural MRI, PET, gene expression and cognitive measures.	Training (70%) and testing (30%)	Correlation analysis	f1-score:0.84
Shu et al., 2021	MCI: 357 AD:154	Structural MRI, CSF, APOE ε4, cognitive measures.	Training (70%) and Testing (30%)	Logistic regression	AUC of 0.814, sensitivity of 0.726, and specificity of 0.798.
Achilleos et al., 2020	NC: 144, AD: 69	Haralick features from hippocampus.	10-fold cross validation	Decision tree and random forests	Accuracy: 0.770
Khan and Zubair, 2020	343 sessions–150 subjects (NC: 72, AD:78)	Structural MRI, cognitive measures, demographics.	Random selection allocation for train, validate and test	Random Forest	Accuracy: 0.868 precision: 0.941 recall: 0.8 AUC: 0.872
Battineni et al., 2020	373 sessions–150 subjects (NC:72, AD:64, MCIc:14)	Structural MRI, cognitive measures, demographics.	10-fold cross validation	Hybrid modeling	Accuracy: 0.980 precision: 0.981 recall: 0.980 ROC: 0.991
Kim et al., 2019	NC:146, AD: 143	Structural MRI.	10-fold cross validation	Principal component and linear discriminant analysis	Accuracy: 75.8%
Spasov et al., 2018	NC: 184, MCI: 409 AD: 192	Structural MRI, APOEε4 cognitive measures, demographics.	10-fold cross validation	Convolution neural network	AUC of 0.925, accuracy: 86%, sensitivity: 87.5% and specificity: 85%

NC, normal control; EMCI, early mild cognitive impairment; LMCI, late mild cognitive impairment; AD, Alzheimer’s disease; MRI, magnetic resonance imaging; PET, positron emission tomography; CSF, cerebrospinal fluid; MMSE, mini mental state examination; CDR, clinical dementia rate; AUC, area under curve; MCIc, mild cognitive impairment converted.

In this study it is hypothesized that through the earlier involvement of entorhinal cortex and hippocampus and by using radiomics, it is likely to detect these microscopic alterations of the disease before atrophy spreads. The use of radiomic features on the entorhinal cortex represents a novelty in the assessment of AD as only in one study has been used before (Leandrou et al., 2020). We aimed to build and validate a radiomics-integrated model through features extracted from both the hippocampus and entorhinal cortex to classify MCI and AD subjects from NC. Only radiomics features were used and the results are compared to other multimodal studies that combined quantitative imaging with other features such as genetics. The paper is organized as follows. The data acquisition is fully described in the section “2. Materials and methods.” In the same section there is a comprehensive description of the data preprocessing and the explainable machine learning model. The results follow in the section “3. Results” and the discussion follow in the section “4. Discussion.” “5. Conclusion” section presents the conclusion over the hypothesis.

## 2. Materials and methods

This is an observational, cross-sectional study. Hence, this study reports its background, methods, and results in line with the STrengthening the Reporting of OBservational studies in Epidemiology (STROBE) reporting guidelines (von Elm et al., 2007). To engage in a transparent way of reporting AI-based studies, this article is also aligned with the Minimum Information about CLinical Artificial Intelligence Modeling (MI-CLAIM) checklist (Norgeot et al., 2020).

### 2.1. The Alzheimer’s Disease Neuroimaging Initiative

Data were acquired from the Alzheimer’s Disease Neuroimaging Initiative (ADNI).^2^ The ADNI was launched in 2003 by the National Institute on Aging, the National Institute of Biomedical Imaging and Bioengineering, the Food and Drug Administration, private pharmaceutical companies and non-profit organizations as a public-private partnership. The goal of the ADNI study is to determine biological biomarkers of AD through neuroimaging, genetics, neuropsychological tests and other measures in order to develop new treatments and monitor their effectiveness and lessen the time of clinical trials.

### 2.2. MRI data

All the subjects had a standardized protocol on 1.5-T MRI units from Siemens Medical Solutions and General Electric Healthcare. MR protocols included high-resolution (typically 1.25 × 1.25 × 1.25 mm^3^ voxels) T1-weighted volumetric 3D sagittal magnetization prepared rapid gradient-echo (MPRAGE) scans. The typical 1.5T acquisition parameters were TR = 2400 ms, minimum full TE, TI = 1000 ms, flip angle = 8°, FOV = 24 cm, with a 256 × 256 × 170 acquisition matrix in the x-, y-, and z-dimensions, yielding a voxel size of 1.25 × 1.25 × 1.2 mm3. MRI data acquisition techniques were standardized across different sites according to ADNI protocol.^3^

### 2.3. Segmentation algorithm and texture analysis

Region of interest segmentation was performed using the Freesurfer image analysis suite (Massachusetts General Hospital, Boston, MA), which is documented and freely available for download online.^4^ The Freesurfer pipeline, conforms the MRI scans to an isotropic voxel size of 1 mm^3^, and the MRI intensity was normalized using the automated N3 algorithm (Sled et al., 1998) followed by skull stripping and neck removal. Details of these have been discussed in previous publications (Fischl et al., 2004, 2002). In brief, this multistep pipeline includes motion correction, automated Talairach transformation, first normalization of voxel intensities, removal of the skull, linear volumetric registration, intensity normalization, non-linear volumetric registration, volumetric labeling, second normalization of voxel intensities, and white matter segmentation. Output includes segmentation of subcortical structures, extraction of cortical surfaces, cortical thickness estimation, spatial normalization onto the FreeSurfer surface template (FsAverage), and parcelation of cortical regions.

Texture features were calculated using KNIME Analytics platform (Berthold et al., 2008). Knime is an open-source bioimage analysis platform which hosts an image processing extension where the user can process and analyze huge amounts of images through workflows. For this study a workflow was build to extract the following Haralick texture features (Haralick et al., 1973): Angular Second Moment (ASM), Contrast, Corelation, Variance, Sum Average, Sum Variance, Entropy and Cluster shade. Their average in four directions (0°, 45°, 90°, 135°) was used.

### 2.4. Subjects

All subjects selected for this study were from standardized data collections^5^ and specifically from the ADNI-1 Complete 2 and 3 year 1.5 Tesla datasets. All data acquired as part of this study are publicly available (see text footnote 2). Enrolled subjects were all between 55 and 90 years of age and each subject was willing, able to perform all test procedures described in the protocol and had a study partner able to provide an independent evaluation of functioning. Overall, 455 subjects were included in the study: 153 NC, 218 MCI and 84 AD as seen in Table 2. According to ADNI protocols, all procedures performed in studies were in accordance with the ethical standards of the institutional and/or national research committee and with the 1964 Helsinki declaration or comparable ethical standards. More details can be found at http://adni.loni.usc.edu/.

**TABLE 2:** Baseline demographics and hippocampal and entorhinal cortex volume.

Variables at baseline	NC (*n* = 194)	MCI (*n* = 284)	AD (*n* = 130)	*p*-value
Sex (M/F)	96/98	181/103	60/70	0.003
Age (mean ± SD)	74.9 (5.2)	71.8 (8.1)	75.0 (7.5)	0.588
MMSE score (mean ± SD)	29 (1.1)	27 (1.4)	23 (2.2)	0.000
Entorhinal cortex volume (mm^3^)	1930 (284)	1719 (384)	1417 (348)	<0.001
Hippocampal volume (mm^3^)	3539 (413)	3243 (461)	2892 (474)	<0.001

NC, normal controls; MCI, mild cognitive impairment; AD, Alzheimer’s disease; MMSE, mini mental state examination; SD, standard deviation.

### 2.5. Cognitive measures

All subjects underwent through clinical and cognitive assessment at the time of baseline scan to determine their diagnosis. Inclusion criteria for NC were: MMSE scores between 24 and 30; CDR of zero; absence of depression, MCI and dementia. Inclusion criteria for MCI were: MMSE scores between 24 and 30; CDR of 0.5; objective memory loss, measured by education adjusted scores on Wechsler Memory Scale Logical Memory II (Elwood, 1991), absence of significant levels of impairment in other cognitive domains and absence of dementia. Inclusion criteria for AD were: MMSE scores between 20 and 26; CDR of 0.5 or 1.0; National Institute of Neurological and Communicative Disorders and Stroke and the Alzheimer’s Disease and Related Disorders Association (NINCDS/ADRDA) criteria for probable AD (McKhann et al., 1984; Dubois et al., 2007). Definitive autopsy-based diagnosis of AD was not possible and detailed description of inclusion/exclusion criteria can be found in the ADNI protocol.^6^

### 2.6. Explainable machine learning and statistical analysis

In the context of explainable ML systems, when a model is built it is important to understand how it is choosing the appropriate features for classification (or prediction). In explainable ML it is estimated how much each feature contributes to the model’s classification. The importance of each feature was evaluated by using the Shapely Addictive exPlanations (SHAP) in terms of Shapley values. A scalable tree boosting system XGBoost ensemble classifier was used which is less prone to overfitting and requires less feature engineering (Chen and Guestrin, 2016).

An individual radiomic feature is generally insufficient to differentiate between the MCI and AD groups. Hence, to achieve a higher likelihood of group separation, a multivariate analysis, which identifies sets of characteristics, was performed. Feature selection methods were applied to avoid overfitting. Initially, a zero or near zero variance filter was used to identify and remove features that were almost constant, and therefore non-informative in the training dataset. Next, a Pearson correlation coefficient (>0.90) was performed to remove redundant features. For the development of this model, first, we split the data in training and test set. The hold-out test set consisted of 30% randomly selected samples from the original data set and the split was stratified so that both train and test sets have the same proportion of labels. A nested 5-fold cross-validation (CV) procedure with an accuracy metric was used to determine the optimal parameters of the learning rate and maximum depth of trees. Randomized grid search is used with 60 iterations is used in order to find the best hyperparameters. The trained model was then applied to the hold-out test set in order to predict the corresponding outcomes. Additionally, accuracy, sensitivity, specificity, FPR: False Positive Rate; FNR: False Negative Rate and area under the receiver operating characteristic (ROC) curve were also calculated, as a measure of the quality of the binary classifications (Table 3).

**TABLE 3:** XGBoost classification performance between groups.

Measure	NC vs. AD		NC vs. MCI		MCI vs. AD	
	**Radiomics**	**Radiomics and cognitive measures**	**Radiomics**	**Radiomics and cognitive measures**	**Radiomics**	**Radiomics and cognitive measures**
Sensitivity	0.799	0.965	0.623	0.764	0.805	0.870
Specificity	0.878	0.907	0.711	0.862	0.582	0.693
Accuracy	0.847	0.946	0.666	0.806	0.719	0.786
Precision	0.822	0.933	0.688	0.881	0.749	0.758
FPR	0.074	0.093	0.142	0.137	0.165	0.306
FNR	0.080	0.034	0.349	0.235	0.118	0.129
f1 Score	0.797	0.949	0.643	0.818	0.766	0.810
ROC	0.910	0.940	0.720	0.880	0.750	0.780

NC, normal controls; MCI, mild cognitive impairment; AD, Alzheimer’s disease; FPR, false positive rate; FNR, false negative rate; ROC, receiver operating characteristic.

**FIGURE 1:**
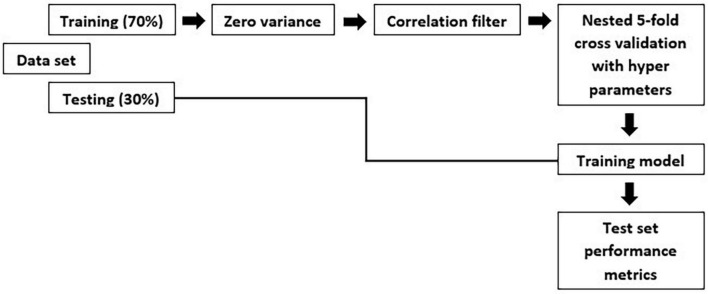
Methodology workflow.

The overall methodology workflow can be seen in Figure 1.

## 3. Results

As mentioned before, in this study we evaluated the importance of each feature by using SHAP values. SHAP value is a measure which shows whether one feature has positive or negative impact on the output and how high affects the model output. A higher SHAP value (higher deviation from the center of the graph) means that the feature value has a higher impact on the prediction for the selected class. Positive SHAP values (points right from the center) are feature values with an impact toward the prediction for the selected class. Negative values (points left from the center) have an impact against classification in this class. F1-score is one of the most important evaluation metrics in ML. It sums up the predictive performance of a model by combining two otherwise competing metrics — precision and recall. Precision is also known as positive predictive value, and recall is also known as sensitivity in diagnostic binary classification.

**FIGURE 2:**
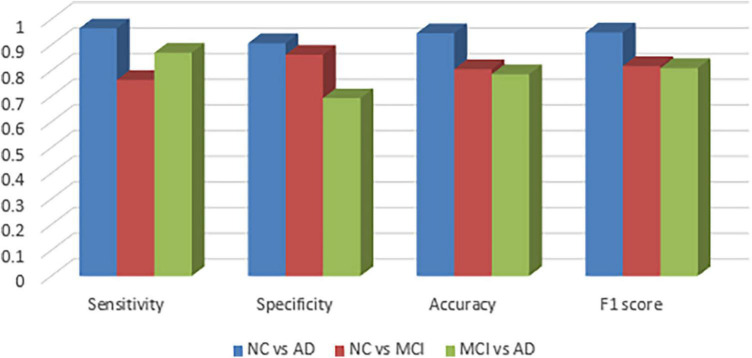
Sensitivity, specificity, accuracy, and f1 scores between the groups.

Figure 2 visualizes sensitivity, specificity, accuracy and f1-score between the groups and as expected, the graphs confirm that NC vs. MCI and MCI vs. AD groups are more difficult to be classified.

Table 3 shows complete evaluation of XGBoost performance between the groups of radiomic features alone and in combination with cognitive measures. To measure the performance of the classification tasks between the groups, sensitivity, specificity, accuracy, precision, False Positive Rate (FPR), False Negative Rate (FNR), f1 score and area under curve (ROC) were calculated. The combination of radiomic and clinical features is most common method in AD research, and in our study XGBoost produced a f1-score of 0.949, 0.818 and 0.810 between NC vs. AD, NC vs. MCI and MCI vs. AD groups, respectively, which is considered to be highly competitive among other studies in the literature. Overall classification accuracy was also very satisfactory deviating from 0.786 to 0.946.

**FIGURE 3:**
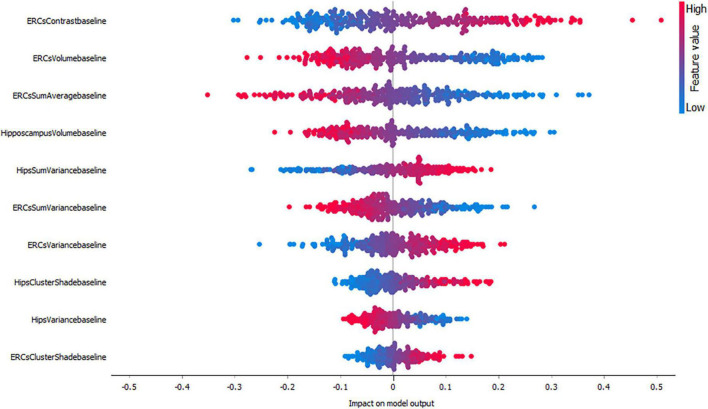
Impact of variables on the classification of NC vs. AD group.

**FIGURE 4:**
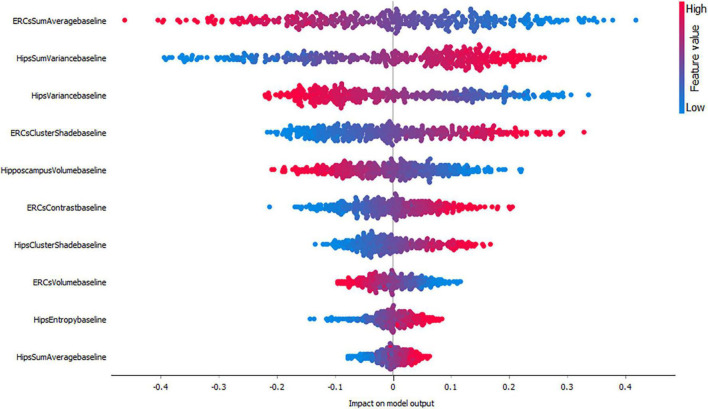
Impact of variables on the classification of NC vs. MCI group.

**FIGURE 5:**
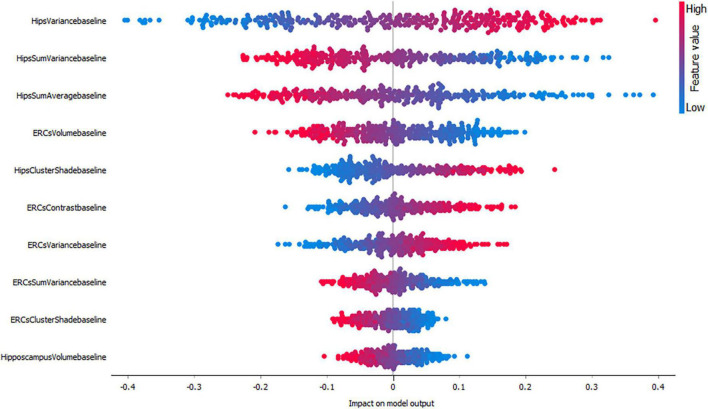
Impact of variables on the classification of AD vs. MCI.

Figures 3–5, depict the summary plots of variables importance in the classification of NC vs. AD, NC vs. MCI and MCI vs. AD. They illustrate the selected number of features that are most important in the classification of NC vs. AD, NC vs. MCI and MCI vs. AD, respectively. For each feature, points in the horizontal axis represent SHAP values.

For the classification of NC vs. AD subjects (Figure 3), it is noticed that the feature which proved to demonstrate the highest positive impact on the model output was the entorhinal cortex contrast. As expected, lower entorhinal cortex and hippocampal volume values, have a positive impact for this group.

When considering the impact of these variables on the model output for the classification of NC vs. MCI (Figure 4) the results indicated a positive impact for low entorhinal cortex sum average values, followed by a positive impact of high hippocampal sum variance values. For this group, both entorhinal cortex and hippocampal volumes, appear to have lower impact on the model as NC and MCI subjects do not have major volume differences.

Finally, the classification of MCI vs. AD (Figure 5) seems to be positively affected by high hippocampal variance measures followed by hippocampal sum variance and sum average where lower values affect the model positively. Interestingly, entorhinal cortex volume seems to have a higher impact compared to hippocampal volume for this group.

## 4. Discussion

In the present study, radiomics signatures from the entorhinal cortex and the hippocampus were used and combined with baseline neuropsychological scales. Then, an XGBoost integrated model was used which has been trained and integrated for the classification of MCI and AD subjects. The model was explained by using the Shapley values produced by the SHAP method. No, genomic data such as apolipoprotein E4 (apoE4) were included in the final model because we wanted to evaluate the performance of radiomic features. The main results are summarized in the Table 3. Our findings indicated that the combination of radiomic features alone or in combination with cognitive measures, could be used for the evaluation of AD.

As in every disease, biomarkers play a crucial role in its early diagnosis. In AD, the most studied biomarkers include biochemical biomarkers such as apoE4 or cerebrospinal fluid (CSF) sample, cognitive tests and neuroimaging markers. However, the application of biochemical markers is not very commonly used due to their interventional collection procedure. On the other hand, cognitive tests will be only applied on patients with symptoms. Therefore, neuroimaging biomarkers especially of those derived from MRI where no ionized radiation is used, are currently the main research focus.

In this study we chose to extract radiomic features from two of the most well studied ROIs in AD, the hippocampus and the entorhinal cortex. Although hippocampus represents the most established ROI used in the assessment of AD, the earlier involvement of the entorhinal cortex was proved by many studies (Gómez-Isla et al., 1997; Juottonen et al., 1999; Galton et al., 2001; Killiany et al., 2002; Busatto et al., 2003; deToledo-Morrell et al., 2004; Tapiola et al., 2008). In two comprehensive reviews (Zhou et al., 2016; Leandrou et al., 2018) the authors concluded that structural changes in the early stages of the disease are more pronounced in the entorhinal cortex. Interestingly, the use of entorhinal cortex texture features in the assessment of AD is very limited in the literature, however, it is the first structure affected by AD. Therefore, in the present study entorhinal cortex texture features were combined with hippocampal texture features and evaluated if there significantly different radiomic features between NC, MCI, and AD subjects.

As the ADNI database is used by many researchers in the assessment of AD, we compared the classification results or our model with those of previous studies. For the classification of NC and AD subjects (Li et al., 2020), used support vector machines (SVM) and RF to verify the efficiency of their model. An average accuracy of 89.7–95.9 and 87.1–90.8% in the validation set and 81.9–89.1 and 83.2–83.7% in the test set, respectively were achieved. Similarly, the study by Jiang et al. (2022) achieved a classification accuracy between NC and AD of 89.85% ± 1.12%. However, in their model apart from MRI radiomic data, cognitive, genetic and PET data were also used. Although our model used only data derived from MRI and cognitive tests it achieved an f1-score of 0.949 and an accuracy of 0.946 for the classification of NC vs. AD subjects. This result is highly competitive among those published in the literature.

In the study by Liu et al. (2018) ML was also used and specifically multiple kernel boosting (MKBoost) algorithm. In their model, included whole brain measures from structural MRI. They achieved an accuracy of 95.65% and a ROC of 0.954 for NC vs. AD, an accuracy of 86.79% and a ROC of 0.826 for NC vs. MCI and an accuracy of 89.63% and an ROC of 0.907 for MCI vs. AD. Their results were similar to our study were a ROC of 0.940, 0.880 and 0.780 were seen between NC vs. AD, NC vs. MCI and MCI vs. AD. However, our study only used 2 structures whereas Liu et al. (2020) used features from the whole brain. In a multimodel deep learning Convolutional Neural Network (CNN) study by Liu et al. (2020), the hippocampus was used for the classification of NC vs. AD, and NC vs. MCI subjects. They achieved an accuracy of 88.9% and an AUC of 92.5%, and an accuracy of 76.2% and an AUC of 77.5%, respectively.

In AD, the classification of MCI subjects is the most challenging as these subjects are not easily identified. These subjects may have decreased memory function beyond the normal level based on a given person’s age and education; however, they do not fulfill the criteria for dementia, as their cognitive function is comparable to NC subjects. Most of the MCI subjects will remain stable even after 10 years of follow-up (Mitchell and Shiri-Feshki, 2009) and only a small percent (10–15%) will progress to AD (Farias et al., 2009). Distinguishing MCI subjects is of great importance and much effort has been put into identifying the MCI subjects that will eventually convert to AD. In this study, for the classification of NC vs. MCI and MCI vs. AD subjects we achieved an f1 score of 0.818 of 0.810 and a ROC of 0.880 and 0.780, respectively. In a similar study, (Shu et al., 2021) for the classification of MCI subjects from AD they achieved an accuracy of 0.814. In another study (Bogdanovic et al., 2022) where XGBoost was also used they achieved a f1-score of 0.840. However, in the aforementioned studies, apart from radiomics, they included other biomarkers such as CSF and/or apoe gene.

Compared to commonly used classification methods such as logistic regression, XGBoost method seems to perform better. Specifically, in the study by Leandrou et al. (2020) where the same database and subjects to this study were used, the classification accuracies between NC vs. AD, NC vs. MCI and MCI vs. AD were 0.914, 0.740 and 0.780, which were lower compared to the XGBoost results of this study. However, although most of the texture features and group of subjects were used, a diagnostic performance comparison between XGBoost and logistic regression is beyond the scope of this study, and should not be criticized only by the aforementioned results. One study that directly compared logistic regression and SVM to XGBoost was made by Suh et al. (2020) and it was found that the use of XGBoost significantly improved the classification compared to the linear Support Vector Machine (SVM) and logistic regression.

Unfortunately, the diagnosis of the disease, still depends on cognitive tests and qualitative imaging assessment. According to the results of this study, quantitative imaging can provide an earlier diagnosis of the disease. However, with quantitative imaging the most major problem of ML is that computers do not explain their predictions which is a barrier to the adoption of ML. What differentiates this study from other ML studies is that the clinician can evaluate the impact of each feature selected by the model. Therefore, the clinical could link a feature used by the model with the history of the patient. Compared to other quantitative imaging features such as from positron emission tomography (PET), MRI lacks of ionizing radiation, therefore, it can be used without any radiation risks. Although amyloid markers such as cerebrospinal fluid (CSF) Amyloid β (Aβ_1–42_) and Aβ PET could detect changes in an earlier stage of the disease, both techniques begin to plateau at the MCI stage where the disease becomes evident (Frisoni et al., 2006). Furthermore, PET studies are not accessible for all subjects, due to several factors such as cost, radiopharmaceutical limitations (availability, targeting amyloid or tau proteins).

Of course, this study has some limitations. First, the sample size could limit the statistical power of the model. Furthermore, only baseline measures were included. Longitudinal measures are very important in AD research to evaluate the overall progress of the subjects, especially of the MCIs. Another, limitation, could be the fact that apart from radiomics and patient demographics, no other biomarkers were included such as, Aβ amyloid, apoE4, CSF sample. However, in this study we wanted to evaluate a radiomics-integrated model only without the aforementioned biomarkers. Future work in AD research should include more participants through multicenter collaboration and datasets.

## 5. Conclusion

Quantitative imaging has shown promising results in the assessment of AD. The results of this study shown that entorhinal cortex and hippocampal texture features can be used as potential biomarkers of the disease and in combination with ML algorithms can provide an earlier diagnosis especially from other quantitative techniques, such as volumetric. One of the most challenging tasks in AD assessment if the identification of MCI subjects. The deep learning-based classification algorithm used in this study accurately differentiated MCI and AD subjects with a relatively high accuracy. It is expected that when radiomic features are combined with other data as well, such as cognitive measures they will perform even better. Furthermore, explainable ML methods can be used to unveil new knowledge to the complexity of AD.

## Data availability statement

The raw data supporting the conclusions of this article will be made available by the authors, without undue reservation.

## Author contributions

SL contributed to the design and implementation of the research and performed the numerical calculations for the suggested experiment. DL and HB contributed to the statistical analysis. KGA and EG contributed to the final form of the manuscript. CSP contributed to the manuscript preparation and revision. All authors discussed the results and commented on the manuscript.

## Footnotes

1
https://www.who.int/news-room/fact-sheets/detail/dementia

2
http://adni.loni.usc.edu/

3
http://adni.loni.usc.edu/methods/documents/mri-protocols/

4
http://surfer.nmr.mgh.harvard.edu/

5
http://adni.loni.usc.edu/methods/mri-analysis/adni-standardized-data/

6
http://adni.loni.usc.edu/methods/documents/

## References

[B1] Achilleos K. G. Leandrou S. Prentzas N. Kyriacou P. A. Kakas A. C. Pattichis C. S. (2020). “Extracting explainable assessments of Alzheimer’s disease via machine learning on brain MRI imaging data, in: 2020 IEEE 20th international conference on bioinformatics and bioengineering (BIBE),” in *Paper Presented at the 2020 IEEE 20th International Conference on Bioinformatics and Bioengineering (BIBE)*, (Cincinnati, OH), 1036–1041. 10.1109/BIBE50027.2020.00175

[B2] Ahmad R. H. M. A. Fakhoury M. Lawand N. (2020). Electromagnetic field in Alzheimer’s Disease: A literature review of recent preclinical and clinical studies. *Curr. Alzheimer Res.* 17 1001–1012. 10.2174/1567205017666201130085853 33256578

[B3] Battineni G. Chintalapudi N. Amenta F. Traini E. (2020). A comprehensive machine-learning model applied to magnetic resonance imaging (MRI) to predict Alzheimer’s disease (AD) in older subjects. *J. Clin. Med.* 9:2146. 10.3390/jcm9072146 32650363 PMC7408873

[B4] Berthold M. R. Cebron N. Dill F. Gabriel T. R. Kötter T. Meinl T. (2008). “KNIME: The Konstanz information miner,” in *Data analysis, machine learning and applications, studies in classification, data analysis, and knowledge organization*, eds Preisach C. Burkhardt H. Schmidt-Thieme L. Decker R. (Berlin: Springer), 319–326.

[B5] Bogdanovic B. Eftimov T. Simjanoska M. (2022). In-depth insights into Alzheimer’s disease by using explainable machine learning approach. *Sci. Rep.* 12:6508. 10.1038/s41598-022-10202-2 35444165 PMC9021280

[B6] Braak H. Braak E. (1997). Frequency of stages of Alzheimer-related lesions in different age categories. *Neurobiol. Aging* 18 351–357. 9330961 10.1016/s0197-4580(97)00056-0

[B7] Busatto G. F. Garrido G. E. J. Almeida O. P. Castro C. C. Camargo C. H. P. Cid C. G. (2003). A voxel-based morphometry study of temporal lobe gray matter reductions in Alzheimer’s disease. *Neurobiol. Aging* 24 221–231. 12498956 10.1016/s0197-4580(02)00084-2

[B8] Chen T. Guestrin C. (2016). “XGBoost: A scalable tree boosting system,” in *Proceedings of the 22nd ACM SIGKDD International Conference on Knowledge Discovery and Data Mining, KDD ’16. Association for Computing Machinery*, New York, NY, 785–794. 10.1145/2939672.2939785

[B9] Chincarini A. Bosco P. Calvini P. Gemme G. Esposito M. Olivieri C. (2011). Local MRI analysis approach in the diagnosis of early and prodromal Alzheimer’s disease. *Neuroimage* 58 469–480. 10.1016/j.neuroimage.2011.05.083 21718788

[B10] deToledo-Morrell L. Stoub T. R. Bulgakova M. Wilson R. S. Bennett D. A. Leurgans S. (2004). MRI-derived entorhinal volume is a good predictor of conversion from MCI to AD. *Neurobiol. Aging* 25 1197–1203. 10.1016/j.neurobiolaging.2003.12.007 15312965

[B11] Dubois B. Feldman H. H. Jacova C. Dekosky S. T. Barberger-Gateau P. Cummings J. (2007). Research criteria for the diagnosis of Alzheimer’s disease: revising the NINCDS-ADRDA criteria. *Lancet Neurol.* 6 734–746. 10.1016/S1474-4422(07)70178-3 17616482

[B12] Elwood R. W. (1991). The Wechsler memory scale—revised: Psychometric characteristics and clinical application. *Neuropsychol. Rev.* 2 179–201. 10.1007/BF01109053 1844708

[B13] Fakhoury M. Piras F. Banaj N. (2021). Editorial: Alzheimer’s disease from a psychiatric perspective: towards new therapeutic guidelines? *Front Psychiatry* 12:782423. 10.3389/fpsyt.2021.782423 34803783 PMC8601228

[B14] Farias S. T. Mungas D. Reed B. R. Harvey D. DeCarli C. (2009). Progression of mild cognitive impairment to dementia in clinic- vs community-based cohorts. *Arch. Neurol.* 66 1151–1157. 10.1001/archneurol.2009.106 19752306 PMC2863139

[B15] Feng F. Wang P. Zhao K. Zhou B. Yao H. Meng Q. (2018). radiomic features of hippocampal subregions in Alzheimer’s disease and amnestic mild cognitive impairment. *Front. Aging Neurosci.* 10:290. 10.3389/fnagi.2018.00290 30319396 PMC6167420

[B16] Fischl B. Salat D. H. Busa E. Albert M. Dieterich M. Haselgrove C. (2002). Whole brain segmentation: automated labeling of neuroanatomical structures in the human brain. *Neuron* 33 341–355. 11832223 10.1016/s0896-6273(02)00569-x

[B17] Fischl B. van der Kouwe A. Destrieux C. Halgren E. Ségonne F. Salat D. H. (2004). Automatically parcellating the human cerebral cortex. *Cereb. Cortex* 14 11–22. 14654453 10.1093/cercor/bhg087

[B18] Folstein M. F. Folstein S. E. McHugh P. R. (1975). Mini-mental state”. A practical method for grading the cognitive state of patients for the clinician. *J. Psychiatr. Res.* 12 189–198. 10.1016/0022-3956(75)90026-6 1202204

[B19] Frisoni G. B. Sabattoli F. Lee A. D. Dutton R. A. Toga A. W. Thompson P. M. (2006). In vivo neuropathology of the hippocampal formation in AD: A radial mapping MR-based study. *Neuroimage* 32 104–110. 10.1016/j.neuroimage.2006.03.015 16631382

[B20] Galton C. Gomez-Anson B. Antoun N. Scheltens P. Patterson K. Graves M. (2001). Temporal lobe rating scale: application to Alzheimer’s disease and frontotemporal dementia. *J. Neurol. Neurosurg. Psychiatry* 70 165–173. 10.1136/jnnp.70.2.165 11160463 PMC1737195

[B21] Gillies R. J. Kinahan P. E. Hricak H. (2016). Radiomics: images are more than pictures. They are data. *Radiology* 278 563–577. 10.1148/radiol.2015151169 26579733 PMC4734157

[B22] Gómez-Isla T. Hollister R. West H. Mui S. Growdon J. H. Petersen R. C. (1997). Neuronal loss correlates with but exceeds neurofibrillary tangles in Alzheimer’s disease. *Ann. Neurol.* 41 17–24. 10.1002/ana.410410106 9005861

[B23] Haralick R. M. Shanmugam K. Dinstein I. (1973). Textural Features for Image Classification. *IEEE Trans. Syst. Man Cybernet. SMC* 3 610–621. 10.1109/TSMC.1973.4309314

[B24] Jack C. R. Shiung M. M. Gunter J. L. O’Brien P. C. Weigand S. D. Knopman D. S. (2004). Comparison of different MRI brain atrophy rate measures with clinical disease progression in AD. *Neurology* 62 591–600. 14981176 10.1212/01.wnl.0000110315.26026.ef PMC2730165

[B25] Jiang J. Zhang J. Li Z. Li L. Huang B. Alzheimer’s Disease Neuroimaging Initiative (2022). Using deep learning radiomics to distinguish cognitively normal adults at risk of Alzheimer’s disease from normal control: An exploratory study based on structural MRI. *Front. Med.* 9:894726. 10.3389/fmed.2022.894726 35530047 PMC9070098

[B26] Juottonen K. Laakso M. P. Partanen K. Soininen H. (1999). Comparative MR analysis of the entorhinal cortex and hippocampus in diagnosing Alzheimer disease. *AJNR Am. J. Neuroradiol.* 20 139–144. 9974069

[B27] Khan A. Zubair S. (2020). an improved multi-modal based machine learning approach for the prognosis of Alzheimer’s disease. *J. King Saud Univ. Comp. Inform. Sci.* 34 2688–2706. 10.1016/j.jksuci.2020.04.004

[B28] Killiany R. J. Hyman B. T. Gomez-Isla T. Moss M. B. Kikinis R. Jolesz F. (2002). MRI measures of entorhinal cortex vs hippocampus in preclinical AD. *Neurology* 58 1188–1196. 10.1212/wnl.58.8.1188 11971085

[B29] Kim J. P. Kim J. Park Y. H. Park S. B. Lee J. S. Yoo S. (2019). Machine learning based hierarchical classification of frontotemporal dementia and Alzheimer’s disease. *Neuroimage Clin.* 23:101811. 10.1016/j.nicl.2019.101811 30981204 PMC6458431

[B30] Leandrou S. Lamnisos D. Mamais I. Kyriacou P. A. Pattichis C. S. (2020). Assessment of Alzheimer’s disease based on texture analysis of the entorhinal cortex. *Front. Aging Neurosci.* 12:176. 10.3389/fnagi.2020.00176 32714177 PMC7351503

[B31] Leandrou S. Petroudi S. Reyes-Aldasoro C. C. Kyriacou P. A. Pattichis C. S. (2018). Quantitative MRI brain studies in mild cognitive impairment and Alzheimer’s disease: A methodological review. *IEEE Rev. Biomed. Eng.* 11 97–111. 10.1109/RBME.2018.2796598 29994606

[B32] Li T.-R. Wu Y. Jiang J.-J. Lin H. Han C.-L. Jiang J.-H. (2020). Radiomics analysis of magnetic resonance imaging facilitates the identification of preclinical Alzheimer’s disease: An exploratory study. *Front. Cell Dev. Biol.* 8:605734. 10.3389/fcell.2020.605734 33344457 PMC7744815

[B33] Liu J. Li M. Lan W. Wu F.-X. Pan Y. Wang J. (2018). Classification of Alzheimer’s Disease Using Whole Brain Hierarchical Network. *IEEE/ACM Trans. Comp. Biol. Bioinform.* 15 624–632. 10.1109/TCBB.2016.2635144 28114031

[B34] Liu M. Li F. Yan H. Wang K. Ma Y. Shen L. (2020). A multi-model deep convolutional neural network for automatic hippocampus segmentation and classification in Alzheimer’s disease. *NeuroImage* 208:116459. 10.1016/j.neuroimage.2019.116459 31837471

[B35] Mayerhoefer M. E. Materka A. Langs G. Häggström I. Szczypiński P. Gibbs P. (2020). Introduction to radiomics. *J. Nucl. Med.* 61 488–495. 10.2967/jnumed.118.222893 32060219 PMC9374044

[B36] McKhann G. Drachman D. Folstein M. Katzman R. Price D. Stadlan E. M. (1984). Clinical diagnosis of Alzheimer’s disease report of the NINCDS-ADRDA work group* under the auspices of department of health and human services task force on Alzheimer’s disease. *Neurology* 34 939–939. 10.1212/WNL.34.7.939 6610841

[B37] Mitchell A. J. Shiri-Feshki M. (2009). Rate of progression of mild cognitive impairment to dementia–meta-analysis of 41 robust inception cohort studies. *Acta Psychiatr. Scand.* 119 252–265. 10.1111/j.1600-0447.2008.01326.x 19236314

[B38] Morris J. C. (1993). The Clinical Dementia Rating (CDR): current version and scoring rules. *Neurology* 43 2412–2414. 10.1212/wnl.43.11.2412-a 8232972

[B39] Norgeot B. Quer G. Beaulieu-Jones B. K. Torkamani A. Dias R. Gianfrancesco M. (2020). Minimum information about clinical artificial intelligence modeling: the MI-CLAIM checklist. *Nat. Med.* 26 1320–1324. 10.1038/s41591-020-1041-y 32908275 PMC7538196

[B40] Petrella J. R. Coleman R. E. Doraiswamy P. M. (2003). Neuroimaging and early diagnosis of Alzheimer disease: a look to the future. *Radiology* 226 315–336. 10.1148/radiol.2262011600 12563122

[B41] Scahill R. I. Schott J. M. Stevens J. M. Rossor M. N. Fox N. C. (2002). Mapping the evolution of regional atrophy in Alzheimer’s disease: Unbiased analysis of fluid-registered serial MRI. *Proc. Natl. Acad. Sci. U.S.A.* 99 4703–4707. 10.1073/pnas.052587399 11930016 PMC123711

[B42] Shu Z.-Y. Mao D.-W. Xu Y.-Y. Shao Y. Pang P.-P. Gong X.-Y. (2021). Prediction of the progression from mild cognitive impairment to Alzheimer’s disease using a radiomics-integrated model. *Ther. Adv. Neurol. Disord.* 14:17562864211029552. 10.1177/17562864211029551 34349837 PMC8290507

[B43] Sled J. G. Zijdenbos A. P. Evans A. C. (1998). A nonparametric method for automatic correction of intensity nonuniformity in MRI data. *IEEE Trans. Med. Imaging* 17 87–97. 10.1109/42.668698 9617910

[B44] Sørensen L. Igel C. Liv Hansen N. Osler M. Lauritzen M. Rostrup E. (2015). Early detection of Alzheimer’s disease using MRI hippocampal texture. *Hum. Brain Mapp.* 37 1148–1161. 10.1002/hbm.23091 26686837 PMC6867374

[B45] Spasov S. Passamonti L. Duggento A. Liò P. Toschi N. (2018). A parameter-efficient deep learning approach to predict conversion from mild cognitive impairment to Alzheimer’s disease. *Neuroimage* 189 276–287. 10.1101/383687 30654174

[B46] Suh C. H. Shim W. H. Kim S. J. Roh J. H. Lee J.-H. Kim M.-J. (2020). Development and validation of a deep learning–based automatic brain segmentation and classification algorithm for Alzheimer disease using 3D T1-weighted volumetric images. *AJNR Am. J. Neuroradiol.* 41 2227–2234. 10.3174/ajnr.A6848 33154073 PMC7963227

[B47] Tapiola T. Pennanen C. Tapiola M. Tervo S. Kivipelto M. Hänninen T. (2008). MRI of hippocampus and entorhinal cortex in mild cognitive impairment: a follow-up study. *Neurobiol. Aging* 29 31–38. 10.1016/j.neurobiolaging.2006.09.007 17097769

[B48] Vaz M. Silvestre S. (2020). Alzheimer’s disease: Recent treatment strategies. *Eur. J. Pharmacol.* 887:173554. 10.1016/j.ejphar.2020.173554 32941929

[B49] Vial A. Stirling D. Field M. Ros M. Ritz C. Carolan M. (2018). The role of deep learning and radiomic feature extraction in cancer-specific predictive modelling: a review. *Transl. Cancer Res.* 7:21823. 10.21037/21823

[B50] von Elm E. Altman D. G. Egger M. Pocock S. J. Gøtzsche P. C. Vandenbroucke J. P. (2007). Strengthening the reporting of observational studies in epidemiology (STROBE) statement: guidelines for reporting observational studies. *BMJ* 335 806–808. 10.1136/bmj.39335.541782.AD 17947786 PMC2034723

[B51] Zhou M. Zhang F. Zhao L. Qian J. Dong C. (2016). Entorhinal cortex: a good biomarker of mild cognitive impairment and mild Alzheimer’s disease. *Rev. Neurosci.* 27 185–195. 10.1515/revneuro-2015-0019 26444348

